# EARLY FLOWERING3 sub-nuclear localization responds to changes in ambient temperature

**DOI:** 10.1093/plphys/kiab423

**Published:** 2021-09-15

**Authors:** James Ronald, Anthony J Wilkinson, Seth J Davis

**Affiliations:** 1 Department of Biology, University of York, Heslington, York YO10 5DD, UK; 2 Department of Chemistry, University of York, Heslington, York YO10 5DD, UK; 3 State Key Laboratory of Crop Stress Biology, School of Life Sciences, Henan University, Kaifeng 475004, China

## Abstract

EARLY FLOWERING3 sub-nuclear localization responds to changes in ambient temperature

Dear Editor,

Plants adapt their development to daily and seasonal ambient temperature fluctuations. Warming triggers a suite of molecular responses that leads to pronounced changes in plant development and architecture (Quint et al., 2016). Collectively, this response is called thermomorphogenesis. The evening complex (EC) is a transcriptional regulatory complex composed of EARLY FLOWERING3 (ELF3), ELF4, and LUX ARRYTHMO (LUX) that has emerged as a hub in the circadian clock and plant development (Nusinow et al., 2011, Herrero et al., 2012; Ezer et al., 2017). The ability of the EC to bind to DNA is temperature-dependent, with warm temperature reducing the association of the EC to DNA (Raschke et al., 2015; Press et al., 2016; Ezer et al., 2017; Silva et al., 2020). However, it is unclear how warm temperature inhibits the DNA binding ability of the EC. Previously, we observed that ELF3 localizes to sub-nuclear structures called foci (Herrero et al., 2012). Impaired localization of ELF3 to foci associated with elevated expression of EC targets (Anwer et al., 2014), suggesting that foci could be sites where the EC binds to DNA and represses gene expression. Therefore, we hypothesized that warm temperature inhibits EC function by reducing the localization of ELF3 to foci.

To test this, first, we investigated whether warm temperature influenced the sub-nuclear localization of ELF3 in Arabidopsis. Using the previously described *35S::YFP:ELF3* (*elf3-4*) line (Herrero et al., 2012), we observed that a 2-h 27°C temperature pulse resulted in fewer and smaller foci in hypocotyl nuclei (Figure 1, A, B, and E). ELF4 is required for ELF3 to localize to foci (Kolmos et al., 2011; Herrero et al., 2012; Anwer et al., 2014) and was also proposed to have a warm temperature specific function in the EC (Jung et al., 2020; Silva et al., 2020). Therefore, we investigated whether ELF4 regulated the sensitivity of ELF3 foci to warm temperature. We introgressed the *35S::YFP:ELF3* line into the *elf3-4/elf4-1* mutant. This line will be referred to as ELF4 (4−), while the original line will be called ELF3 (4+). The localization of ELF3 (4−) to foci was impeded at 22°C (Figure 1C and this was further reduced by a 27°C pulse (Figure 1, D and E). However, the relative change in the number of foci for ELF3 (4−) in response to the 27°C pulse was similar to ELF3 (4+) (Figure 1F). Together, warm temperatures suppress the localization of ELF3 to foci and ELF4 has a limited role within this.

**Figure 1:**
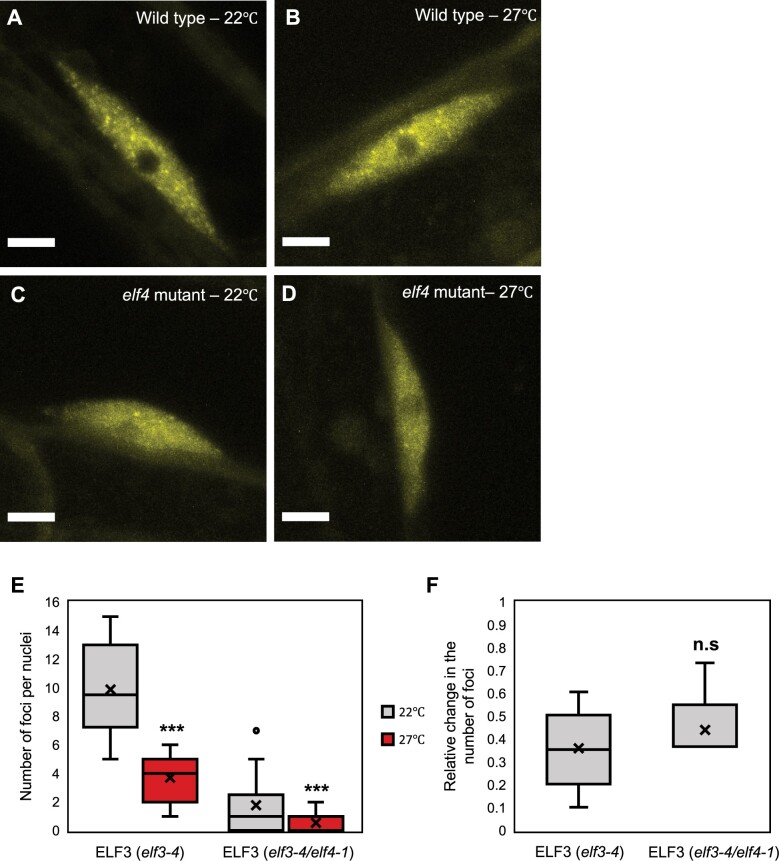
Elevated temperature reduces the association of ELF3 to foci in hypocotyl nuclei. The localization of ELF3 at dusk in hypocotyl nuclei of (A and B) *35S::YFP:ELF3 elf3-4* (parental type) or (C and D) *35S::YFP:ELF3 elf3-4/elf4-1* (*elf4* mutant) plants. Images were taken at (A, C) 22°C or (B, D) after a 2-h 27°C pulse started at ZT6 (short-day 8/16 photoperiods). E, Number of foci per nucleus under the respective treatment. F, Relative change in the number of ELF3 foci following a temperature pulse in the *elf3-4* or *elf3-4/elf4-1* mutant. Data were relative to the respective genotype at 22°C. For *elf3-4/elf4-1*, nuclei with no foci were removed from this calculation. Images were collected on two occasions, with a minimum of 10 images analyzed in total. For both (E and F) the center line within the box defines the median, with the cross defining the mean. The limits of the box define the upper and lower quartiles, while the whiskers extend 1.5× the interquartile range. Significance was determined by a T test: n.s = no significance, ***P < 0.001. Scale bars are 5 μm.

To understand if the response of ELF3 foci to temperature was tissue-dependent, we investigated the effect of a 27°C pulse on foci formation in root nuclei. As in hypocotyl nuclei, a 27°C pulse suppressed the localization of ELF3 (4+) to foci in root nuclei and these foci were smaller and less bright than at 22°C (Figure 2, A and B). There was no substantial change in the magnitude of effect caused by the 27°C pulse between the tissue types, with ELF3 (4+) foci reduced by 62% and 60% in hypocotyl and root nuclei, respectively. Therefore, ELF3 foci do not have a tissue-dependent response to warm temperature.

**Figure 2:**
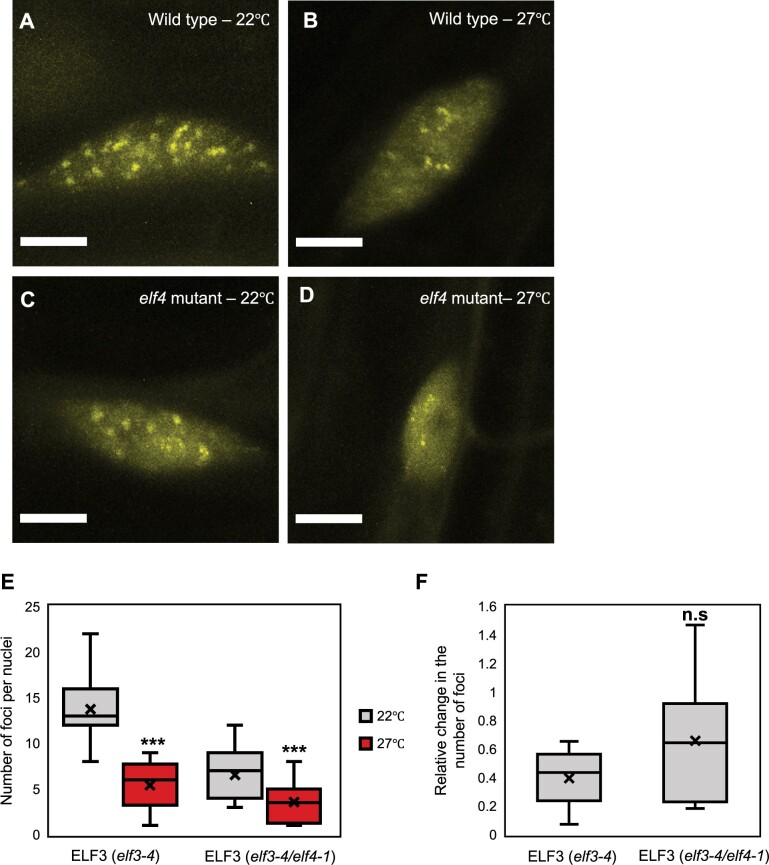
A 27°C pulse inhibits the association of ELF3 to foci in root nuclei. The localization of ELF3 at dusk in root nuclei of (A and B) *35S::YFP:ELF3 elf3-4* (wild type) or (C and D) *35S::YFP:ELF3 elf3-4/elf4-1* (*elf4* mutant). Images were taken at (A and C) 22°C or (B and D) after a 2-h 27°C pulse started at ZT6 (short-day 8/16 photoperiods). E, Number of foci per nuclei under the respective treatment. F, Relative change in the number of ELF3 foci following a temperature pulse in the *elf3-4* or *elf3-4/elf4-1* background. Data were relative to the respective genotype at 22°C. Images were collected on two occasions, with a minimum of 10 images analyzed in total. For both (E and F) the center line within the box defines the median, with the cross defining the mean. The limits of the box define the upper and lower quartiles, while the whiskers extend 1.5× the interquartile range. Significance was determined by a T test: n.s = no significance, ***P < 0.001. Scale bars are 5 μm.

As ELF4 protein moves from shoot to root tissue, and this movement is temperature sensitive (Chen et al., 2020), we examined the requirement of ELF4 in regulating the thermal responsiveness of ELF3 foci in root nuclei. As with ELF3 (4+), ELF3 (4−) localized to foci in root nuclei and these foci were larger and brighter than those in hypocotyl nuclei (Figures 1C and 2C). The phenotypic effect of the *elf4-1* mutation on ELF3 foci abundance was weaker in root nuclei, with foci only reduced by 52% compared to an 81% reduction in hypocotyl nuclei at 22°C. ELF3 (4−) foci in root nuclei were also reduced by a 27°C temperature pulse (Figure 2, D and E). However, this effect was more variable and on average weaker than the response of ELF3 (4+) foci to the 27°C pulse (Figure 2F). As with ELF3 (4+), ELF3 (4−) foci appeared smaller and less bright after a 27°C pulse in root nuclei (Figure 2, C and D). Combined, ELF4 does not have a critical role in buffering ELF3 foci against warming temperatures in either hypocotyl or root nuclei.

The decrease in foci number following a 27°C pulse could reflect changes in the nuclear accumulation of ELF3. To investigate this, we measured ELF3 (4+) and ELF3 (4−) nuclear signal at 22°C and 27°C in hypocotyl and root nuclei. As we have reported previously (Herrero et al., 2012), ELF4 is required for proper nuclear accumulation of ELF3 (Supplemental Figure 1). ELF3 (4−) had a lower nuclear accumulation than ELF3 (4+) in hypocotyl and root nuclei. The 27°C pulse also strongly reduced the nuclear accumulation of ELF3 (4+) in hypocotyl nuclei (Supplemental Figure 1A). Furthermore, there was a cumulative effect of the 27°C pulse and the *elf4-1* mutation on the nuclear accumulation of ELF3 in hypocotyl nuclei. A similar response to the 27°C pulse was seen in root nuclei for both ELF3 (4+) and ELF3 (4−) (Supplemental Figure 1B). As with hypocotyl nuclei, the 27°C pulse and the *elf4-1* mutation had a cumulative effect on ELF3 nuclear accumulation (Supplemental Figure 1B). The reduced nuclear accumulation of ELF3 at 27°C is consistent with a recent report that ELF3 is degraded by B-BOX ZINC FINGER PROTEIN 18 (BBX18) and XB3 ORTHOLOG 1 IN ARABIDOPSIS THALIANA (XBAT31) and XBAT35 at warm temperatures (Zhang et al., 2021a, 2021b).

In summary, the localization of ELF3 to foci is suppressed by warm temperature and ELF4 does not seem to regulate this process. Thus, ELF4 must stabilize the function of the EC at warm temperatures through a separate mechanism (Silva et al., 2020). As the localization of ELF3 to foci is associated with increased transcriptional activity of ELF3, a reduction in foci may contribute to the weaker EC function at warm temperatures (Kolmos et al., 2011; Ezer et al., 2017). We also highlight a recent report that observed ELF3 localizing to sub-nuclear structures called speckles in response to warming (Jung et al., 2020). Direct comparisons are complicated because of the different genetic resources and experimental conditions used, but in the supplemental text, we discuss why our results may diverge from the work of Jung *et al.* (2020).

## Supplemental Data

The following materials are available in the online version of this article.

**Supplemental Figure S1.** Elevated temperature reduces the nuclear accumulation of ELF3.

**Supplemental Text.**

## Supplementary Material

kiab423_Supplementary_Data

## Notes

The author responsible for distribution of materials integral to the findings presented in this article in accordance with the policy described in the Instructions for Authors (https://academic.oup.com/plphys/pages/general-instructions) is: Seth J. Davis (seth.davis@york.ac.uk).

## References

[kiab423-B1] Anwer MU , Boikoglou E, Herrero E, Hallstein M, Davis AM, Velikkakam James G, Nagy F, Davis SJ (2014) Natural variation reveals that intracellular distribution of ELF3 protein is associated with function in the circadian clock. eLife 3**:** e02206 10.7554/eLife.02206 PMC4071560 24867215

[kiab423-B2] Chen WW , Takahashi N, Hirata Y, Ronald J, Porco S, Davis SJ, Nusinow DA, Kay SA, Mas P (2020) A mobile ELF4 delivers circadian temperature information from shoots to roots. Nat Plants 6**:** 416–426 32284549 10.1038/s41477-020-0634-2 PMC7197390

[kiab423-B3] Ezer D , Jung J-H, Lan H, Biswas S, Gregoire L, Box MS, Charoensawan V, Cortijo S, Lai X, Stöckle D, et al (2017) The evening complex coordinates environmental and endogenous signals in Arabidopsis. Nat Plants 3**:** 17087–17087 28650433 10.1038/nplants.2017.87 PMC5495178

[kiab423-B4] Herrero E , Kolmos E, Bujdoso N, Yuan Y, Wang M, Berns MC, Uhlworm H, Coupland G, Saini R, Jaskolski M, et al (2012) EARLY FLOWERING4 recruitment of EARLY FLOWERING3 in the nucleus sustains the Arabidopsis circadian clock. Plant Cell 24**:** 428–443 22327739 10.1105/tpc.111.093807 PMC3315225

[kiab423-B5] Jung J-H , Barbosa AD, Hutin S, Kumita JR, Gao M, Derwort D, Silva CS, Lai X, Pierre E, Geng F, et al (2020) A prion-like domain in ELF3 functions as a thermosensor in Arabidopsis. Nature 585**:** 256–260 32848244 10.1038/s41586-020-2644-7

[kiab423-B6] Kolmos E , Herrero E, Bujdoso N, Millar AJ, Tóth R, Gyula P, Nagy F, Davis SJ (2011) A reduced-function allele reveals that EARLY FLOWERING3 repressive action on the circadian clock is modulated by phytochrome signals in Arabidopsis. Plant Cell 23**:** 3230–3246 21908721 10.1105/tpc.111.088195 PMC3203447

[kiab423-B7] Nusinow DA , Helfer A, Hamilton EE, King JJ, Imaizumi T, Schultz TF, Farré EM, Kay SA (2011) The ELF4-ELF3-LUX complex links the circadian clock to diurnal control of hypocotyl growth. Nature 475**:** 398–402 21753751 10.1038/nature10182 PMC3155984

[kiab423-B8] Press MO , Lanctot A, Queitsch C (2016) PIF4 and ELF3 Act Independently in Arabidopsis thaliana Thermoresponsive Flowering. PLoS One 11**:** e0161791–e0161791 27564448 10.1371/journal.pone.0161791 PMC5001698

[kiab423-B9] Quint M , Delker C, Franklin KA, Wigge PA, Halliday KJ, Van Zanten M ( 2016) Molecular and genetic control of plant thermomorphogenesis. Nat Plants 2**:** 15190 27250752 10.1038/nplants.2015.190

[kiab423-B10] Raschke A , Ibañez C, Ullrich KK, Anwer MU, Becker S, Glöckner A, Trenner J, Denk K, Saal B, Sun X, et al (2015) Natural variants of ELF3 affect thermomorphogenesis by transcriptionally modulating PIF4-dependent auxin response genes. BMC Plant Biol 15**:** 197 26269119 10.1186/s12870-015-0566-6 PMC4535396

[kiab423-B11] Silva CS , Nayak A, Lai X, Hutin S, Hugouvieux V, Jung J-H, López-Vidriero I, Franco-Zorrilla JM, Panigrahi KCS, Nanao MH, et al (2020) Molecular mechanisms of evening complex activity in Arabidopsis. Proc Natl Acad Sci USA 117: 6901–6909 32165537 10.1073/pnas.1920972117 PMC7104408

[kiab423-B12] Zhang LL , Shao YJ, Ding L, Wang MJ, Davis SJ, Liu JX (2021) XBAT31 regulates thermoresponsive hypocotyl growth through mediating degradation of the thermosensor ELF3 in Arabidopsis. Sci Adv 7: eabf4427 33962946 10.1126/sciadv.abf4427 PMC8104893

[kiab423-B13] Zhang LL , Wei L, Tang YY, Davis SJ, Liu JX (2021) The E3 ligase XBAT35 mediates thermoresponsive hypocotyl growth by targeting ELF3 for degradation in Arabidopsis. J Integr Plant Biol 63: 1097–1103 33963671 10.1111/jipb.13107

