# Supplementary material for: EARLY FLOWERING3 sub-nuclear localization responds to changes in ambient temperature
Source: Plant Physiol. 2021 Sep 15;187(4):2352–5. doi: 10.1093/plphys/kiab423 (PMC8644450; doi:10.1093/plphys/kiab423)
Supplement: kiab423_Supplementary_Data [file kiab423_supplementary_data.pdf]

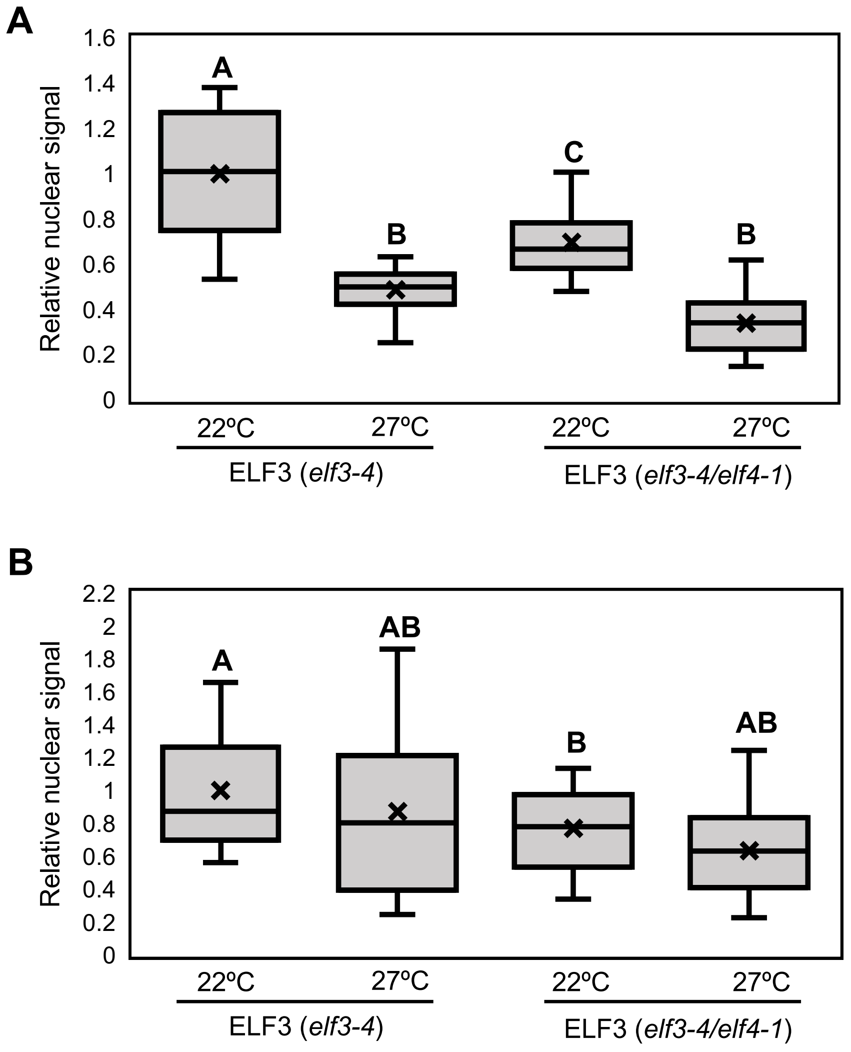

Supplemental Figure S1. Elevated temperature reduces the nuclear accumulation of ELF3.

The relative nuclear signal of ELF3 in (**A**) hypocotyl or (**B**) root nuclei. Nuclear signal was quantified using the images collected for the foci counts of figure 1 and figure 2, respectively. The nuclear signal was made relative to the nuclear signal of ELF3 in the *elf3-4* background at 22ºC for each respective tissue type. Images were collected on two separate occasions with a combined n of 12 or more images analyzed for each respective genotype and temperature treatment. For both (A, B) the center line within the box defines the median, with the cross defining the mean. The limits of the box define the upper and lower quartiles, while the whiskers extend 1.5x the interquartile range. Significance was calculated using a one-way ANOVA with a tukey-HSD post-hoc test. Different letters signify a significance of $p < 0.05$.

Supplemental Text

Discussion

There are multiple possible explanations for why our results on temperature suppression of ELF3 foci formation diverge from the results of earlier work where it was reported that temperature promotes ELF3 speckle formation (Jung *et al.,* 2020[1]). Firstly, Jung *et al.,* (2020) used a native promoter of *ELF3*, while here a *35S* promoter was used to drive *ELF3* expression. We consider it unlikely that the use of a *35S* promoter has driven aberrant foci formation as previous work that used a native promoter of *ELF3* also observed foci at ambient temperatures[2]. Alongside the promoter, the fluorochrome used also varied between the two pieces of work. Here, we used a N-terminal eYFP tag (pENSG-YFP[3] vector), while the work of Jung *et al.,* (2020) used a C-terminus GFP fluorochrome tag[1]. The YFP sequence in pENSG-YFP has the following encoded mutations compared to GFP: S65G/S72A/T203Y/H231L. The pENSG-YFP vector has been widely used for analyzing protein localization *in planta* before and there have been no reports of this vector promoting nuclear-localized proteins to form sub-nuclear structures[3,4,5]. Therefore, we consider it unlikely that the eYFP tag is promoting the localization of ELF3 to foci.

Further differences in the genetic constructs between the two pieces of work include the source of the ELF3 sequence. Our *ELF3* sequence was cloned from the Arabidopsis accession Ws-2[6], while the *ELF3* sequence of Jung *et al.,* (2020) was cloned from Col-0[1]. Ws-2 and Col-0 ELF3 sequences have differing poly-Q lengths, and the poly-Q stretch of ELF3 was reported to be part of the prion-like domain that contributes to temperature sensing[1,7,8]. Hence, differences in the length of the poly-Q domain could plausibly result in temperature-dependent changes in the behavior of Ws-2 and Col-0 ELF3. However, previous work failed to highlight a role for the poly-Q domain in ELF3 temperature signaling[9] and instead was proposed to contribute to the general function of ELF3 activity[10]. Additionally poly-Q length variation is not seen in all species[1]. Therefore, the significance of the variation in poly-Q

length in causing temperature-dependent differences in the behavior of ELF3 Ws-2 and Col-0 is uncertain.

Alongside differences in the structural composition of the genetic resource used, biological reasons could potentially explain the divergence in results. ELF3 has been reported to co-localize to sub-nuclear structures with multiple proteins and these proteins have different temporal dynamics in gene expression and protein stability[6,11-13]. As these temporal protein-protein interactions influence the cellular and sub-cellular localization of ELF3[2,6,13] , changes in the timing of the warming-pulse application could subsequently influence or change the effect warming-pulse has on the sub-nuclear localization of ELF3. Here, we applied the warming-pulse at ZT6 (2-hours before dusk, see supplementary methods), while it is unreported when the warming -pulse were applied in the work of Jung *et al.*, (2020). We applied our warming-pulse at ZT6 because of the dusk protein-accumulation profile of ELF3 and the other EC components under a short-day photoperiod[14]. However, ELF3 also interacts and co-localizes with morning-phased proteins TZP and phyB[12]. As the activity and cellular localization of phyB is also temperature sensitive[15,16], it is possible that time-of-day changes in when the warming-pulses are applied could influence the subsequent sub-cellular response of ELF3. Further work is needed to understand the causative factor resulting in these divergence results.

Materials and Methods

Plant lines

All Arabidopsis lines used in this report were in the Ws-2 background. The *elf3-4 LHY::LUC*, *elf4-1 LHY::LUC*, *elf3-4/elf4-1* and *35S::YFP:ELF3 LHY::LUC* (*elf3-4*) lines have all been described previously[6]. The *35S::YFP:ELF3 (elf3-4/elf4-1*) line was generated in the course of this work by crossing the *35S::YFP:ELF3 LHY::LUC* into the *elf3-4/elf4-1* mutant. All lines were genotyped using previously published primer sequences[6].

Confocal microscopy

The Leica Zeiss 710 confocal laser scanning microscope with Plan-Apochromat 63x/1.4 Oil DIC M27 objective and Zen 2011 SP4 confocal software (Leica) was used to collect images. Arabidopsis seedlings were submerged in deionized water on clear white slides. For all constructs, the YFP fluorochrome was excited at 514 nm and emission detected between 525-615 nm. The pinhole was set to airy one for all constructs. The same laser setting was used for all images collected during this work, regardless of the mutant background: laser power = 4%, master gain = 695, digital gain = 2.6 and digital offset = 23.40. All images were collected as Z-stacks, with a pixel size of 512x512 and a Z-stack slice depth of 0.4 μm.

Foci counts and nuclear signal measurements

Arabidopsis seeds were surfaced sterilized and plated onto 1x Murashige and Skoog (MS) plates (0.25% w/v sucrose, 0.5 g/L MES and 1.5% phytoagar) and stratified for three days. After stratification, seedlings were transferred to a short-day (8 hours light, 16 hours of dark) growth chamber with 85 μmol/m^{2}/s^{1} of light and a constant temperature of 22ºC for 7 days. At ZT6 (two hours before dusk) on day 7, seedlings to be heat pulsed were transferred to a 27ºC growth chamber (~25 μmol/m^{2}/s^{1} white light) for two hours. The control plates were kept at 22ºC but moved to a chamber with a lower light intensity (~25 μmol/m^{2}/s^{1}white light, similar spectral composition as the 27ºC chamber) to eliminate any effect of light on foci formation. At the end of the two-hour heat pulse, seedlings were imaged between ZT8

and ZT9. Seedlings not being imaged were kept at 27ºC in the dark to ensure no rapid reversion in foci dynamics occurred at cooler temperatures.

Foci were manually counted from compiled Z-stacks projected as a 2.5D min-max image in the Zen 2011 SP4 software. These counts were then validated by manually scoring each image of the compiled Z-stack for foci. To measure the nuclear signal, collected images were compiled as Z-stacks in the Zen 2011 SP4 confocal software and then imported into imageJ (V.1.52k) for measurements. Nuclear signal was measured as integrated density. In total, a minimum of 10 images were analyzed for the foci count and nuclear signal measurements. Images were captured on multiple occasions, with similar results observed on the separate microscopy sessions.

Supplemental References:

1) Jung *et al.,* 2020. Nature. DOI: 10.1038/s41586-020-2644-7
2) Anwer *et al.,* 2014. eLife. DOI 10.7554/eLife.02206
3) Laubinger *et al.,* 2006. Development. DOI: 10.1242/dev.02481
4) Zhu *et al.,* 2008. Plant Cell. DOI: 10.1105/tpc.107.056580
5) Rishmawi *et al.,* 2014. Plant Physiol. DOI: 10.1104/pp.113.233775
6) Herrero *et al.,* 2012. Plant Cell. DOI: 10.1105/tpc.111.093807
7) Tajima *et al.,* 2007. Plant Biotechnology. DOI: 10.5511/plantbiotechnology.24.237
8) Undurraga *et al.,* 2012. PNAS. DOI: 10.1073/pnas.1211021109
9) Press *et al.,* 2016. PLOS ONE. DOI: 10.1371/journal.pone.0161791
10) Press & Queitsch. 2017. Genetics. DOI: 10.1534/genetics.116.193359
11) Yu *et al.,* 2008. Molecular Cell. DOI 10.1016/j.molcel.2008.09.026
12) Kaiserli *et al.,* 2015. Developmental Cell. DOI: 10.1016/j.devcel.2015.10.008
13) Kolmos *et al.,* 2011. Plant Cell. DOI: 10.1105/tpc.111.088195
14) Nusinow *et al.,* 2011. Nature. DOI:10.1038/nature10182
15) Jung *et al.,* 2016. Science. DOI: 10.1126/science.aaf6005
16) Hahm *et al.,* 2020. Nature Comms. DOI: 10.1038/s41467-020-15526-z.
